# Silent Circulation of St. Louis Encephalitis Virus Prior to an Encephalitis Outbreak in Cordoba, Argentina (2005)

**DOI:** 10.1371/journal.pntd.0001489

**Published:** 2012-01-31

**Authors:** Luis Adrian Díaz, Guillermo Albrieu Llinás, Ana Vázquez, Antonio Tenorio, Marta Silvia Contigiani

**Affiliations:** 1 Laboratorio de Arbovirus, Instituto de Virología “Dr. J. M. Vanella”, Facultad de Ciencias Médicas, Universidad Nacional de Córdoba, Córdoba, Argentina; 2 Instituto de Investigaciones Biológicas y Tecnológicas (IIByT), Consejo Nacional de Investigaciones Científicas y Técnicas (CONICET), Córdoba, Argentina; 3 Investigador Asistente, Carrera del Investigador Científico, CONICET, Ministerio de Ciencia y Tecnología, Buenos Aires, Argentina; 4 Laboratorio de Arbovirus, Centro Nacional de Microbiología, Instituto de Salud Carlos III, Majadahonda, Spain; Centers for Disease Control and Prevention, United States of America

## Abstract

St. Louis encephalitis virus is a complex zoonoses. In 2005, 47 laboratory-confirmed and probable clinical cases of SLEV infection were reported in Córdoba, Argentina. Although the causes of 2005 outbreak remain unknown, they might be related not only to virological factors, but also to ecological and environmental conditions. We hypothesized that one of the factors for SLE reemergence in Córdoba, Argentina, was the introduction of a new SLEV genotype (SLEV genotype III), with no previous activity in the area. In order to evaluate this hypothesis we carried out a molecular characterization of SLEV detections from mosquitoes collected between 2001 and 2004 in Córdoba city. A total of 315 mosquito pools (11,002 individuals) including 12 mosquitoes species were analyzed. Overall, 20 pools (8 mosquitoes species) were positive for SLEV. During this study, genotypes II, V and VII were detected. No mosquito pool infected with genotype III was detected before the 2005 outbreak. Genotype V was found every year and in the 8 sampled sites. Genotypes II and VII showed limited temporal and spatial activities. We cannot dismiss the association of genotype II and V as etiological agents during the outbreak. However, the silent circulation of other SLEV strains in Córdoba city before the 2005 outbreak suggests that the introduction of genotype III was an important factor associated to this event. Not mutually exclusive, other factors such as changes in avian hosts and mosquitoes vectors communities, driven by climatic and environmental modifications, should also be taken into consideration in further studies.

## Author Summary

The St. Louis encephalitis is a complex zoonoses in the New World. In South America (Argentina and Brazil), SLE is an emerging arbovirosis. SLEV reemerged in Argentina during 2002 and, since then, outbreaks have been reported in 2005, 2006, 2010 and 2011. During the 2005 outbreak two SLEV genotype III strains were isolated. Although the causes of the 2005 outbreak remain unknown, they might be related not only to virological factors, but also to changes in the structure and dynamics of vectors and/or avian amplifying hosts' populations and environmental conditions. We hypothesized that one of the factors for SLE reemergence in Córdoba, Argentina, was the introduction of a new SLEV genotype, with no previous activity in the area. No mosquitoes were detected infected with genotype III during this four-year study, even 10 months before the outbreak. The silent circulation of other SLEV strains in Córdoba city before the 2005 outbreak suggests that the introduction of genotype III was an important factor associated to this event. Not mutually exclusive, other factors such as changes in avian hosts and mosquitoes vectors communities, driven by climatic and environmental modifications, should also be taken into consideration in further studies.

## Introduction

The St. Louis encephalitis (SLE), caused by the homonymous virus (SLEV, genus *Flavivirus*, family *Flaviviridae*), is a complex zoonoses in the New World [1]. In South America, SLE is an emerging arbovirosis, with febrile illness and encephalitis cases reported in Argentina and Brazil [2], [3]. SLEV reemerged in the central region of Argentina during 2002 [2] and, since then, outbreaks have been reported in Córdoba (2005) [4], Entre Rios (2006), Buenos Aires (2010) and San Juan provinces (2011) [5].

SLEV shows biological and molecular variability among strains isolated throughout its geographic distribution [6]–[8]. Based on complete Envelope gene sequencing, SLEV strains can be classified into 8 lineages or genotypes (I–VIII) [9]. According to a phylogeographic analyses, genotypes I and II are prevalent in the USA, while the others were found only in countries from Central and South America [9]–[11]. Exceptionally, genotype V strains were recently isolated in Florida [12]. SLEV strains circulating in Argentina were clustered with genotype III (79V-2533 –year 1978-, CbaAr-4005, CbaAr-4006 – year 2005-), V (78V-6507 – year 1978-), both isolated from *Culex* spp. Mosquitoes, and VII (CorAn-9124, CorAn-9275 – year 1966-), isolated from rodents [9], [13].

In USA, the transmission cycles of SLEV are maintained by *Culex* mosquito vector species (*Culex quinquefasciatus*, *Cx. tarsalis* and *Cx. nigripalpus*) and Columbiformes (Mourning doves - *Zenaida macroura*) and Passeriformes (House finches - *Carpodacus mexicanus*, House sparrows - *Passer domesticus*) bird host species. Humans and mammals represent a dead-end host for the virus. Although it is widely distributed in the American continent, its ecology is scarcely known outside the USA [1].

Available data in Argentina suggests that *Cx. quinquefasciatus* mosquito would act as a main vector, while Picui ground dove (*Columbina picui*) and Eared dove (*Zenaida auriculata*) would be important hosts in urban and rural environments [13]–[16]. However, an alternative transmitting rodent-mosquito cycle was postulated in Argentina for genotype VII SLEV strains (CorAn-9124, CorAn-9275), isolated from rodents [14].

SLEV reemerged in the central region of Argentina in 2002 [2]. In 2005, 47 laboratory-confirmed and probable clinical cases of SLEV infection, including nine fatalities, were reported in the central Córdoba Province [4]. During this outbreak two SLEV genotype III strains were isolated [13]. This was the first SLEV-induced encephalitis outbreak reported in South America. Although the causes of 2005 outbreak remain unknown, they might be related not only to virological factors, but also to changes in the structure and dynamics of vectors and/or avian amplifying hosts populations, and environmental conditions. We hypothesized that one of the factors for SLE reemergence in Córdoba, Argentina was the introduction of a new SLEV genotype, with no previous activity in the area. In order to evaluate this hypothesis we carried out a molecular characterization of SLEV detections from mosquitoes collected between 2001 and 2004 in Córdoba city.

## Methods

### Collection sites

**Figure 1:**
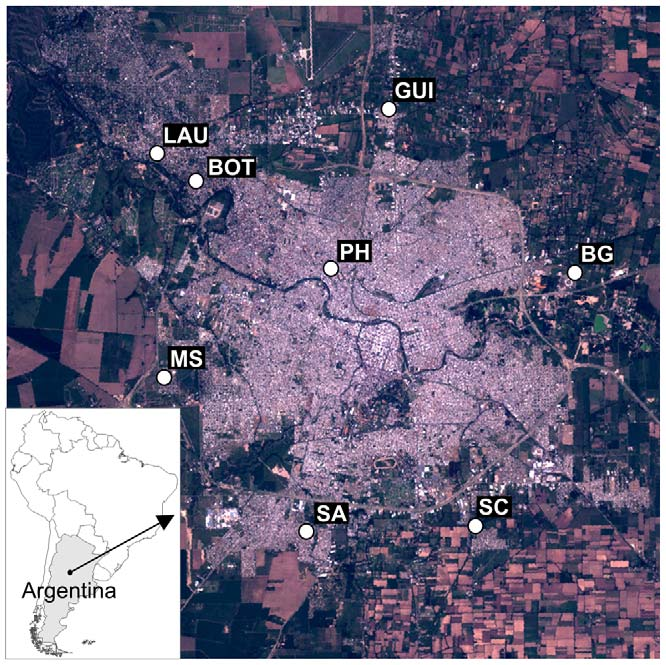
Sampling sites where mosquito collections were carried out during 2001–2004. LAU: Libre del Ambiente University; BOT: Botanic Garden; GUI: Guiñazú; PH: Pediatric Hospital; BG: Bajo Grande; MS: Military School; SA: San Antonio; SC: San Carlos.

Mosquito collections were carried out during 2001 to 2004 in the city of Córdoba (31°24′30″S, 64°11′02″O) (Córdoba Province, Argentina), just before the 2005 SLE local outbreak. The city is situated at 450 m over sea level and its extension covers 576 km^2^, of which 37.2% is urbanized. The population is 1,330,023 inhabitants (http://200.51.91.231/censo2010/). The area belongs to the phytogeographic region of the Espinal, Chaqueño Domain, intensively modified by human activities (urbanization, agriculture, cattle, and industry). The city is surrounded by land crops (soy, fruit tree), industries and autochthonous shrubs patches isolated. The climate is mild without warm winters and with water deficit, in spite of its relatively high precipitation levels (750 and 800 mm), due to high evapotranspiration. A total of 8 collection sites were selected based on accessibility, owners' authorizations and previous evidence of mosquitoes abundance; most of them are located in peripheral areas of the city of Córdoba (Figure 1).

### Sample collection

Mosquitoes were collected using CDC light traps (supplemented with dry ice) and chicken and rabbit baited can traps. Three light traps and 2 baited traps were set up and maintained in each site during 2 nights per season. Traps remained active during 18:00 until 09:00. The trapping schedule is detailed in Table 1. Collected mosquitoes were transported alive in refrigerated containers to the laboratory. Individuals were identified on a chill table and sorted by species, sex, collection date and site, and non-engorged and engorged status. Mosquitoe pools were homogenized using pestles and mortars in minimum essential medium (MEM) supplemented with 10% fetal bovine sera (FBS), 1% gentamicine and 1% Fungizone. Pools containing 1–25 mosquitoes were homogenized in 1 ml of MEM and those containing 25–50 mosquitoes were added 2 ml of MEM. Homogenates were centrifuged at 11,400 g during 30 min at 4°C for decontamination. Supernatants were stored in tubes at −70°C until utilization.

**Table 1:** Mosquito collection carried out in Córdoba during summer and fall season between 2001–2004.

Year	Month	Site	Trap
2001	November	Bajo Grande	CDC light trap
	December	Botanical Garden	Chicken can trap
		C. San Antonio	Rabbit can trap
		Aviation School	
		Pediatric Hospital	
		Guiñazú	
		U. Libre del Ambiente	
2002	January	Bajo Grande	CDC light trap
	February	Botanical Garden	Chicken can trap
	March	C. San Antonio	Rabbit can trap
	April	C. San Carlos	
	November	Aviation School	
	December	Pediatric Hospital	
		Guiñazú	
		U. Libre del Ambiente	
2003	January	Bajo Grande	CDC light trap
	February	Botanical Garden	Chicken can trap
	March	C. San Carlos	
	April	Pediatric Hospital	
		Guiñazú	
2004	January	Bajo Grande	CDC light trap
	February	Pediatric Hospital	
	March	C. San Carlos	
	April	Guiñazú	

### Flavivirus and SLEV molecular detection and sequencing analyses

#### RT-Nested PCR

Viral RNA was extracted from 150 µl of the mosquito pool homogenates using 750 µl of Trizol Reagent (Invitrogen BRL, Life Technologies, Rockville, MD), following the manufacturer indications. Reverse transcription was carried out with Moloney Murine Leukemia virus reverse transcriptase (MMLV, Promega, Madison, WI, USA) with Random Hexamer Primers (Promega, Madison, WI, USA) following the manufacturer indications. The cDNAs obtained from pools were used to amplified a 143 bp fragment from NS5 Flavivirus protein and 234 pb fragment from the Envelope gene of SLEV using Green Go Taq Reagents (Promega, Madison, WI, USA), as described previously [17], [18]. Positive RT-Nested SLEV specific PCR mosquito homogenates were subjected to viral isolation attempts. VERO (Green monkey kidney cells) cells monolayer were inoculated with 100 µl mosquito homogenates and checked daily for cytopathic effect.

#### Sequencing and phylogenetic analyses

All PCR products obtained from mosquito pools were purified using the QIAquick gel extraction kit (Qiagen, Valencia, CA, USA), submitted to direct nucleotide sequencing reaction using Big Dye Terminator v3.1 Cycle kit (Applied Biosystem), and analyzed in an automatic sequencer. The obtained sequences were submitted to BLASTn 2.2.19 -Basic Local Alignment Search Tool- (http://blast.ncbi.nlm.nih.gov) to detect homologies among Flaviviruses and SLEV sequences available in the data base. A phylogenetic analysis of the SLEV alignment including 134 partial Envelope gene sequences was performed using neighbor joining (NJ) in MEGA v5 [19]; the proportion of differences between the sequences was considered as the distance measure (*p* distance). The NJ analysis was bootstrapped 10,000 replications.

### Viral isolation attempts

RT PCR SLEV positive mosquito pools were subjected to viral isolation. One hundred µl of mosquito homogenate were inoculated onto 24 hs VERO cells monolayers, incubated for 60 min at 37°C, and observed daily for cythopatic effect. After 7 day post inoculation, blind passages were realized.

## Results

A total of 315 mosquito pools (11,002 individuals) including 12 mosquitoes species were analyzed. Overall, 20 pools (8 mosquitoes species) were positive for SLEV (Table 2). The BLASTn search and the phylogenetic analyses were performed using a 212 bp fragment of the Envelope protein. Viral isolation attempts were unsuccessful due to probable multiple freeze/thaws cycles during the process of mosquito homogenates.

**Table 2:** SLEV RT-PCR positive mosquito pools collected in Córdoba city, Argentina between 2001 and 2004.

Pool	Especie	GenBank	Month/Year	Site	Genotype
CbaAr 1-12	*Cx. quinquefasciatus*	FJ361863	02/02	Bajo Grande	V
CbaAr 2-45	*Ae. scapularis*	FJ361867	11/02	Botanic Garden	V
CbaAr 2-25	*Cx. quinquefasciatus*	FJ361872	11/02	Bajo Grande	V
CbaAr 2-51	*An. albitarsis*	FJ361877	12/02	Bajo Grande	V
CbaAr 2-71	*Cx. apicinus*	FJ361861	12/02	Pediatric Hosp.	V
CbaAr 2-80	*Cx. interfor*	FJ361880	12/02	C. San Carlos	VII
CbaAr 2-52	*Cx. quinquefasciatus*	FJ361873	12/02	Bajo Grande	V
CbaAr 2-35	*Ps. ferox*	FJ361876	12/02	Guiñazú	V
CbaAr 2-108	*Ae. aegypti*	FJ361869	01/03	Botanic Garden	V
CbaAr 2-85	*Ae. albifasciatus*	FJ361881	01/03	Bajo Grande	II
CbaAr 2-161	*Ae. scapularis*	FJ361878	02/03	Bajo Grande	VII
CbaAr 2-252	*Cx. quinquefasciatus*	FJ361862	02/03	Bajo Grande	V
CbaAr 2-248	*Cx. quinquefasciatus*	FJ361866	02/03	Pediatric Hosp.	V
CbaAr 2-191	*Ae. albifasciatus*	FJ361860	03/03	Guiñazú	V
CbaAr 2-267	*Ae. albifasciatus*	FJ361879	03/03	Guiñazú	VII
CbaAr 2-210	*Cx. interfor*	FJ361864	03/03	Pediatric Hosp.	V
CbaAr 2-190	*Cx. quinquefasciatus*	FJ361875	03/03	Guiñazú	II
CbaAr 2-292	*Ae. albifasciatus*	FJ361874	04/03	C. San Carlos	V
CbaAr 2-305	*Ae. albifasciatus*	FJ361865	04/03	C. San Carlos	V
CbaAr 3-80	*Ae. scapularis*	FJ361868	02/04	C. San Carlos	V

**Figure 2:**
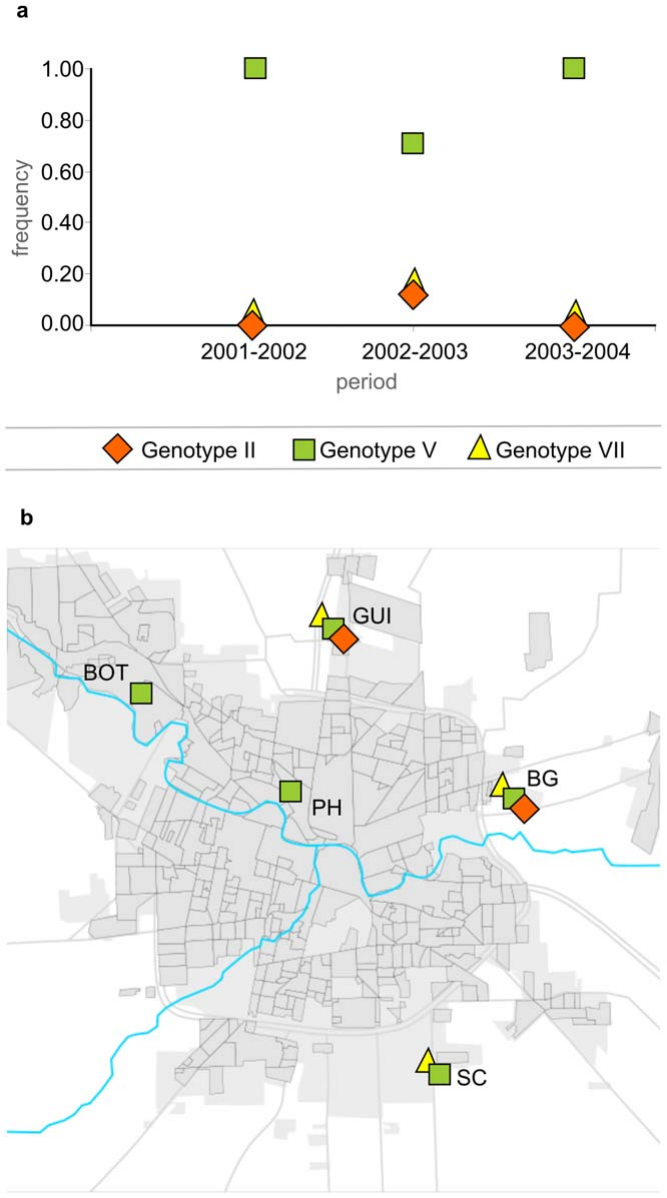
SLEV genotype activity in mosquitoes collected in Córdoba city between 2001–2004. **a**) SLEV genotypes temporal distribution. **b**) SLEV genotypes geographical distribution. LAU: Libre del Ambiente University; BOT: Botanic Garden; GUI: Guiñazú; PH: Pediatric Hospital; BG: Bajo Grande; MS: Military School; SA: San Antonio; SC: San Carlos. White circles represent sampled sites without SLEV activity detected during the study period.

**Figure 3:**
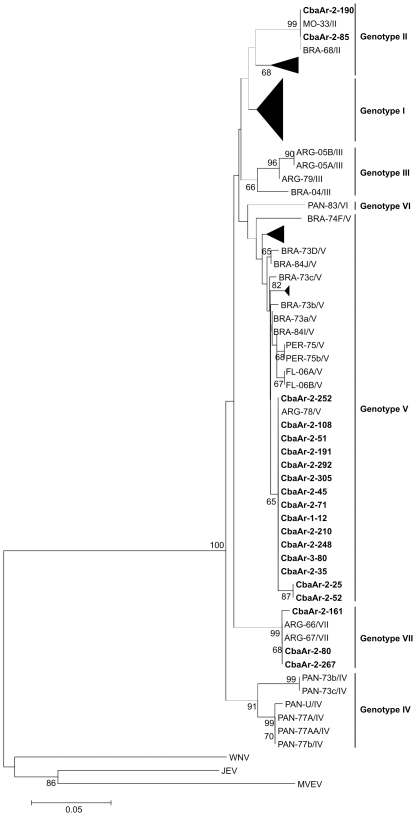
Phylogenetic Neighbor Joining analysis of SLEV. In bold strains detected in mosquitoes during our study in Córdoba, Argentina. Percentage of replicate trees in which the associated taxa clustered together in the bootstrap test (10 000 replicates) is shown next to the branches. The scale bar indicates substitutions per site.

Based on the classification proposed by Kramer and Chandler [9], three different SLEV genotypes (II, V, and VII) were detected in Córdoba city before the encephalitis human outbreak associated to SLEV genotype III (Figure 2). Genotype V was detected in 5 of the 8 collection sites during the 4 years (Figure 3) and it was found in the 8 infected mosquito species collected in our study (*Aedes aegypti*, *Anopheles albitarsis*, *Ae. albifasciatus*, *Ae. scapularis*, *Culex apicinus*, *Cx. interfor*, *Cx. quinquefasciatus*, *Psoropohora ferox*) (Table 2). Sequences belonging to genotype V share a 100% of homology with SLEV strain 78V6507 (AF205481). This genotype was previously isolated from *Cx. quinquefasciatus* mosquitoes in Santa Fe province in 1978 [20].

Genotypes II and VII were detected only sporadically during the study. However, this is the first report of SLEV genotype II in Argentina. The nucleotide sequence corresponding to this genotype share 100% homology with SLEV strains SPAn-11916 (EF117302), was isolated in 1968 from rodents collected in Sao Paulo (Brazil), and Parton-MSI-7 (EF158070) was isolated from ill humans in Missouri (USA) in 1933. Only 2 positive pools (*Ae. albifasciatus* and *Cx. quinquefasciatus* mosquitoes) collected in Bajo Grande and Guiñazú in 2003 proved to be infected with this genotype in our study (Figure 3). Sequences clustering with Genotype VII have a 100% of homology with SLEV strains CorAn-9124 (EF158063) and CorAn-9275 (EF158068) isolated in 1966. Although genotype VII has been isolated from small rodents (*Calomys musculinus*, *Mus musculus*) in the province of Córdoba [14], there was no previous report of its activity neither in mosquitoes nor in the capital city.; it was detected in 3 sites (Guiñazú, Bajo Grande, San Carlos) during 2002 and 2003 (Table 2, Figure 3). Mosquitoes infected with genotype VII were identified as *Ae. albifasciatus*, *Ae. scapularis* and *Cx. interfor*.

## Discussion

Three SLEV genotypes circulated simultaneously in Córdoba city between 2001 and 2004. None of these genotypes are related to the SLEV Genotype III CbaAr-4005 strain isolated during the encephalitis human outbreak that occurred later in 2005 [13].

The most prevalent variant was genotype V, genetically related to SLEV 78V-6507 strain isolated in Santa Fe province in 1978 [20]. Our results confirm the endemicity of SLEV in Córdoba city during 2001–2005. The sustained activity of genotype V in Bajo Grande, Botanical Garden, Guiñazú, Pediatric Hospital, and San Carlos between 2001 and 2004 (Figure 1) indicates local genotype persistence and probable overwintering of SLEV activity in this temperate area. *Culex* mosquitoe abundance decreases drastically during winter, so vector transmission to vertebrates would not be maintained during this season. Supporting endemicity of SLEV, Flores et al. [21] detected vertical transmission of SLEV genotype V in local *Culex* mosquito populations under laboratory conditions. We believe that this mechanism would satisfactory explain the maintenance of some viral variants until the next favorable season for mosquito vector proliferation.

The presence of a predominant genotype could be evidence for a higher viremogenic capacity of this strain in avian hosts. Higher viremias would enhance the transmission by mosquitoes, increasing viral circulation and expanding the distribution of this variant. In fact, SLEV genotype V (strain 78V-6507) developed higher viremias in birds than genotype III and VII strains [7]. SLEV not only requires the ability to infect avian hosts with highly enough viremias to be deemed infectious to mosquitoes, but it also needs to be able to disseminate the mosquito mid-gut and enter the salivary glands. This ecological and evolutionary feature should not be unattended, indeed.

In a different way, genotypes II and VII showed limited temporal and spatial activities (Figure 1). Powers et al. [22] pointed out that arboviruses maintained by a rodents-mosquitoes cycle show limited and constrained geographic distributions, which agree with the limited dispersion capacity observed in rodents. In fact, Genotype VII SLEV strains (CorAn-9124, CorAn-9275) were isolated only from small rodents in Córdoba province [14], which suggests that mammals are the actual hosts for these strains. Moreover, the 3 mosquito species (*Ae. albifasciatus*, *Ae. scapularis* and *Cx. interfor*) infected with Genotype VII frequently feed on mammals hosts [20], [23]–[25]. Our data support the hypothesis that some SLEV strains are being maintained through alternative rodents-mosquitoes transmission cycles [14].

Analyzing temporal and geographic patterns of SLEV in Texas by molecular techniques, Chandler et al. [26] detected the presence of dominant and limited strains fluctuating in space and time. This activity pattern could be the result of multiple intermittent virus introductions by birds from neighboring regions [27]. Other flavivirus such as Japanese encephalitis (JEV) and West Nile virus (WNV) showed similar dynamics [28], [29]. These dynamics are characterized by the introduction/persistence of certain genotypes and the presence/absence of clinical cases in the study area. Another possibility is that SLEV genotype III had been silently present in Córdoba prior to 2001. Although a Dengue virus has different ecological requirements compared with JEV, SLEV and WNV, it has been demonstrated that some serotypes can remain silent for many years causing periodic epidemics [30].

During 2005, a human encephalitis outbreak was caused by SLEV in Córdoba city [4], and two genotype III strains were isolated [13]. Molecular characterization determined that both variants are closely related to SLEV strain 79V-2533, isolated 27 years ago in Santa Fe province [13]. This evidence supports the hypothesis that the introduction of a more virulent genotype could have caused the mentioned outbreak. In a recent study, Diaz et al. [31] compared epidemic (CbaAr-4005) and non-epidemic (79V-2533) genotype III SLEV strains. The epidemic variant produced higher viremias in House sparrows (*Passer domesticus*) than the non-epidemic strain. According to this, the epidemic strain (CbaAr-4005) appears to broaden the number of avian species that are likely to be competent amplifying hosts relative to the non-epidemic 79V-2533 strain [31].

The introduction of new strains and the extension of their geographic distributions are factors that can cause the emergence and reemergence of flaviviruses in different regions [32]. For example, the introduction, spread and establishment of WNV in America and Japanese Encephalitis Virus in Australasia and the annual introduction of SLEV strains in the USA (California state) [27]. During our four-year study a non-genotype III SLEV strain was detected. One year prior to the outbreak a total of 2,093 mosquitoes were analyzed and only one mosquito pool was positive (genotype V) (Table 1). The results here exposed suggest that genotype III was introduced in Córdoba city a few months before the outbreak and it could be one of the factors contributing to the outbreak.

SLEV strains show biological and molecular variability [6]–[9]. Strains belonging to genotypes I, II, III, and V showed pathogenicity in mice and Rhesus monkeys [6]. During SLEV human encephalitis outbreaks in the USA, genotypes I and II were frequently isolated [9]. Spinsanti et al. [2] confirmed one human encephalitis case in Córdoba concomitant with our detection of Genotype V in the same area during the same year (Pediatric Hospital, 2002 – Figure 1). Although genotype III was detected in mosquitoes collected around the encephalitis human cases during the 2005 outbreak, we cannot dismiss the association of genotype II and V as etiological agents during the outbreak. However, the silent circulation of other SLEV strains in Córdoba city before the 2005 outbreak suggests that the introduction of genotype III was an important factor associated to this event. Not mutually exclusive, other factors such as changes in avian hosts and mosquitoes vectors communities, driven by climatic and environmental modifications, should also be taken into consideration in further studies.
